# Therapeutic Monitoring of Patients With Hereditary Tyrosinemia Type 1—A Belgian Monocentric Experience

**DOI:** 10.1002/jmd2.70062

**Published:** 2026-01-07

**Authors:** Anne‐Sophie Adam, Lionel Marcélis, David Fage, Elise Mathieu, Aurélie Empain, Céline Dufour, Frédéric Cotton, Corinne de Laet

**Affiliations:** ^1^ Department of Clinical Chemistry, Laboratoire Hospitalier Universitaire de Bruxelles Université Libre de Bruxelles Brussels Belgium; ^2^ Laboratory of Paediatric Research Université Libre de Bruxelles Brussels Belgium; ^3^ Nutrition and Metabolic Clinic Brussels University Hospital, Academic Children Hospital Queen Fabiola, Université libre de Bruxelles Brussels Belgium; ^4^ European Reference Network for Hereditary Metabolic Disorders (MetabERN) Member Padova Italy

**Keywords:** dried blood spot, hereditary tyrosinemia type‐1, nitisinone, succinylacetone, therapeutic monitoring

## Abstract

Hereditary tyrosinemia type I (HT‐1) is a rare metabolic disorder treated by NTBC, requiring careful therapeutic and nutritional monitoring. While follow‐up traditionally relies on urinary succinylacetone, plasma NTBC and plasma amino acids, dried blood spot (DBS) methods have introduced alternative monitoring options. However, the optimal biochemical monitoring remains debated. This study evaluated the clinical utility of NTBC measurements compared with established biomarkers in HT‐1. In this retrospective single‐centre study, we analysed biological data from 12 HT‐1 patients treated with NTBC over 6 years. We analysed correlations between NTBC, succinylacetone, δ‐aminolevulinic acid (δ‐ALA) and alpha‐fetoprotein concentrations, and compared tyrosine and phenylalanine levels in DBS and plasma. Succinylacetone suppression in both urine and blood was achieved across a broad range of NTBC concentrations, suggesting that blood succinylacetone is a more reliable marker of metabolic control than NTBC levels. Elevated urinary δ‐ALA levels were observed in some samples despite unquantifiable succinylacetone, indicating that succinylacetone may not fully reflect neurological risk. NTBC concentrations showed limited correlation with alpha‐fetoprotein, reinforcing the continued need for imaging in hepatocellular carcinoma surveillance. DBS measurement of tyrosine and phenylalanine displayed variable biases relative to plasma, particularly for tyrosine, highlighting the challenges of using DBS for nutritional monitoring. While NTBC remains central in the treatment of HT‐1 patients, its blood concentrations offer limited added value for long‐term monitoring. Focusing on succinylacetone measurement, along with δ‐ALA and alpha‐fetoprotein to evaluate neurological and hepatic risks, is recommended. Plasma remains the preferred matrix for amino acids monitoring. Larger multi‐centre studies are needed to confirm these findings.

## Key Points

- Succinylacetone is a better indicator of metabolic control than NTBC blood levels.
- NTBC concentration adds limited value for long‐term follow‐up.
- δ‐aminolevulinic acid and alpha‐fetoprotein remain essential complementary biomarkers.
- Dried blood spots are useful but have limitations for nutritional monitoring.
- A monitoring strategy centred on succinylacetone, with δ‐aminolevulinic acid and alpha‐fetoprotein as needed, is more informative than relying on NTBC blood levels.

## 1 Introduction

Hereditary tyrosinemia type I (HT‐1, OMIM 276700) is a rare autosomal recessive disorder of tyrosine (Tyr) catabolism, with an estimated global incidence of 1:100 000 live births, though it is more frequent in regions with founder effects [1, 2].

HT‐1 is caused by a deficiency of the enzyme fumarylacetoacetate hydrolase (FAH, EC 3.7.1.2), the final enzyme in the Tyr catabolism pathway [3]. This deficiency leads to the accumulation of toxic intermediates (i.e., maleylacetoacetate, fumarylacetoacetate, succinylacetoacetate, and succinylacetone (SA)), responsible for the hepatic, renal, and neurological damages that may coexist in these patients [4, 5, 6, 7, 8].

Before the 1990s, treatment options were limited to dietary restrictions of Tyr and phenylalanine (Phe), and liver or combined liver‐kidney transplantation [9]. In 1992, the discovery of the efficacy of nitisinone (NTBC) in the treatment of HT‐1 revolutionised the management and prognosis of this disease. NTBC is a potent competitive inhibitor of 4‐hydroxyphenylpyruvate dioxygenase (HPPD), thus preventing the accumulation of the toxic metabolites and therefore the clinical manifestations of HT‐1 [10]. However, it causes Tyr to accumulate, requiring lifelong dietary restriction of Tyr and Phe [8].

In view of the major complications of HT‐1 [4, 9], effective therapeutic monitoring is of paramount importance and includes therapeutic drug monitoring (TDM) and nutritional monitoring.

The TDM of NTBC could be achieved either by measuring NTBC blood concentrations (Pharmacokinetic (PK) monitoring) or by monitoring biomarkers of response to NTBC (Pharmacodynamic (PD) monitoring). The latter is the approach used by most clinicians by measuring urinary and/or blood SA levels. In theory, NTBC is not a drug that could benefit from PK monitoring [11, 12, 13], but recent recommendations suggest giving the minimum dose of NTBC to achieve NTBC blood concentrations of 40–60 μmol/L [8]. Although this range is based on limited data and may require revision [9, 14, 15, 16, 17, 18, 19]. Moreover, while there is a clear consensus on the monitoring of alpha‐fetoprotein (AFP) levels in blood, the best biomarker for hepatocellular carcinoma (HCC), no recommendations exist for the monitoring of delta‐5‐aminolevulinic acid (δ‐ALA) in HT‐A patients, the most used biomarker for acute pseudo‐porphyric crisis, with significant disparities between laboratories [4, 8, 20].

Nutritional monitoring targets plasma Tyr levels of 200–600 μmol/L and Phe levels of 20–80 μmol/L [4, 8]. However, standardisation of Tyr and Phe measurement in DBS remains lacking, with conflicting data on Tyr plasma‐to‐DBS correlation [21].

Despite guidelines, there is no consensus on the optimal NTBC monitoring approach, and practices vary by laboratory capabilities [19]. Nutritional monitoring guidelines remain limited to plasma values, restricting the adoption of home‐based DBS sampling, which could offer practical benefits.

This study aimed to investigate (i) whether recommended NTBC concentrations are necessary for SA suppression, (ii) if correlations between NTBC and/or SA levels with different biochemical markers exist and (iii) the feasibility of DBS monitoring for NTBC, SA, Tyr and Phe. In particular, we try to assess whether NTBC or SA alone may serve as the central monitoring parameter and whether all traditional biological parameters are necessary.

## 2 Materials, Subjects and Methods

### 2.1 Subjects

This is a retrospective study of the patients with HT‐1 followed in the Nutrition and Metabolism Unit of the Queen Fabiola Children's University Hospital (HUDERF, Brussels, Belgium) between January 2019 and June 2024. All patients were treated with NTBC and had a Tyr/Phe restricted diet with specific amino acids (AA) supplementation. Dietary modifications were made according to the Tyr target concentration in plasma (200–600 μmol/L), and Phe was supplemented to maintain concentrations above 20 μmol/L. This study was approved by the HUDERF ethics committee and conducted in accordance with the Declaration of Helsinki (as revised in 2013).

### 2.2 Clinical Samples

DBS, lithium heparin (LH) plasma, serum and urine samples from each HT‐1 patient were collected at the same time, during their clinical follow‐up visits at HUDERF. For correlation studies, paired samples from each patient at each medical visit were analysed as independent samples.

### 2.3 Laboratory Analysis

For DBS analysis, NTBC levels were determined by liquid chromatography tandem mass spectrometry (LC–MS/MS) while Phe, Tyr and SA were determined by direct loop injection into an MS/MS system. Details on these methods can be found in Supporting Information. Urinary δ‐ALA levels were determined with the ClinEasy Complete Kit (RECIPE Chemicals + Instruments GmbH, Munich, Germany). AFP concentrations were determined on a Cobas 8000 (Roche Diagnostics, Brussels, Belgium). Urinary SA levels were determined by an external laboratory by gas chromatography–mass spectrometry (GC–MS). Plasma amino acids Phe and Tyr were also determined by an external laboratory by ion exchange chromatography and post‐column derivatisation with ninhydrin in a Biochrom 30 amino acid analyser (Biochrom Ltd., Cambridge, UK).

### 2.4 Statistical Analysis

A descriptive analysis of all variables was performed. For each variable, the distribution was determined using the Shapiro–Wilk test. The Kruskal‐Wallis test followed by Dunn's multiple comparison post‐test was used to compare parameters separated into three or more groups. When two groups were compared, the Mann–Whitney test was used. Results were presented as mean ± standard error of the mean (SEM). A Spearman correlation test was used to check for associations between certain variables. To compare Phe and Tyr concentrations between DBS and plasma, Passing and Bablok regression analyses were performed. In addition, Bland Altman tests were performed to visualise the relationship between bias and concentration. All significance testing was two‐tailed and a *p*‐value (*p*) below 0.05 was considered statistically significant.

All statistical analyses were performed using GraphPad Prism software v10.4.1 and the XLSTAT premium module (version 2023.3.1.1416) in Microsoft Excel v16.95.1.

## 3 Results

### 3.1 Subjects

Twelve HT‐1 patients were identified (seven men, five women) from nine families. Nine patients were of consanguineous parentage. The median duration of treatment was 17 years [2–28 years] and the median age at last follow‐up was 18 years [3–28 years]. No patient developed hepatocellular carcinoma (HCC) during the study period, and none required liver transplantation. However, some patients still had borderline AFP and one patient had a remodelled liver on imaging. The median, mean, minimum and maximum values for each parameter and each patient are summarised in Table S1.

### 3.2 Therapeutic Monitoring

#### 3.2.1 SA

As SA was undetectable in all our collected urine samples, only blood SA levels measured on DBS were investigated in this study.

**FIGURE 1:**
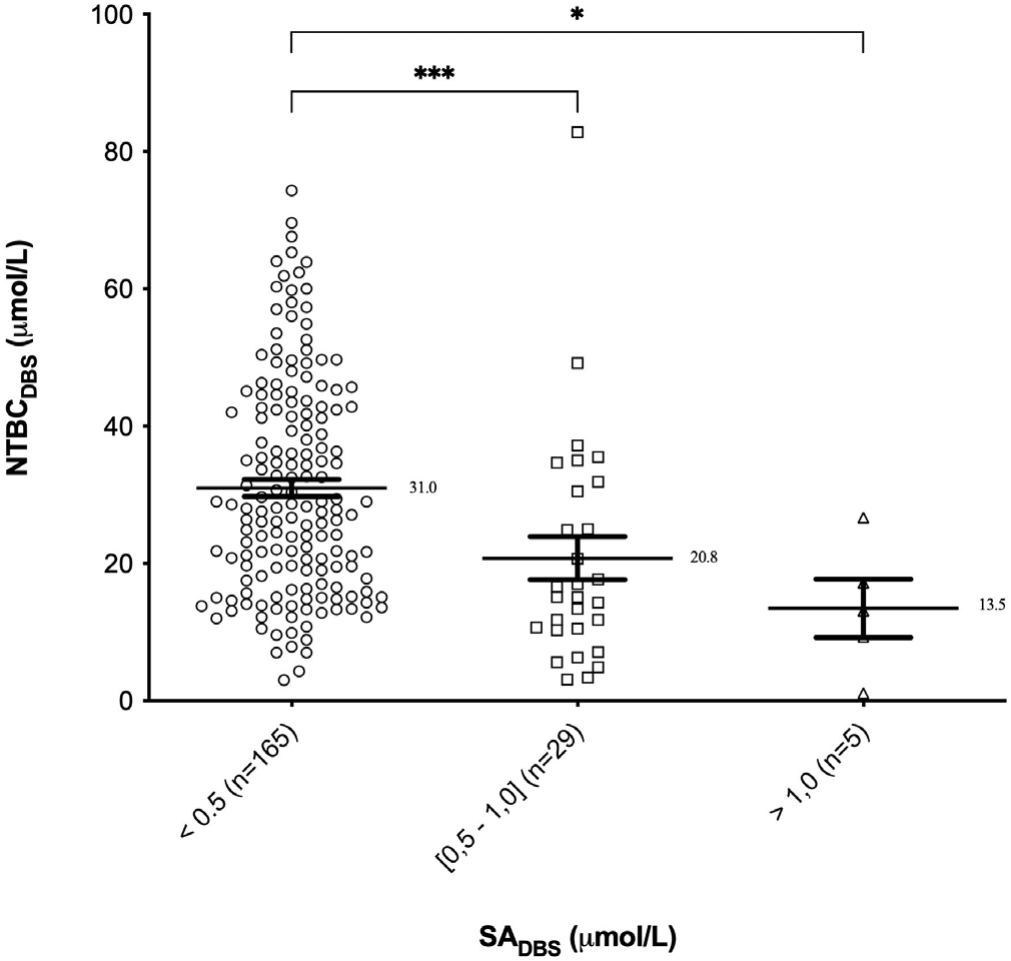
NTBC concentrations according to SA concentrations in DBS (mean ± SEM). (*p ≤ 0.05; ***p ≤ 0.001).

A total of 197 DBS samples were analysed. The NTBC‐SA paired samples were classified according to their SA concentration into three groups: < 0.5 μmol/L, between 0.5 and 1.0 μmol/L and > 1.0 μmol/L. These groups were created for two reasons: (i) the limit of quantitation (LOQ) for our method is 0.5 μmol/L, so anything below this threshold is not accurately quantifiable; and (ii) the thresholds of 0.5 μmol/L and 1.0 μmol/L are used by our clinicians to adapt the dosage of NTBC according to their internal procedures. The mean NTBC concentration in the 165 paired samples with SA below 0.5 μmol/L was 31.0 μmol/L. Twenty‐nine paired samples had SA between 0.5 and 1.0 μmol/L and the mean of NTBC in this group was 20.8 μmol/L. Finally, five paired samples had SA on DBS above 1.0 μmol/L and the mean concentration in this group was 13.5 μmol/L. The Kruskal–Wallis test showed a significant difference between the three groups (*p* = 0.0001). Dunns' multiple comparison test showed that NTBC concentrations were significantly higher in samples with SA below 0.5 μmol/L compared with the other two groups (Figure 1). A correlation analysis between these SA and NTBC in DBS was not feasible, as the majority of our paired samples had SA levels in DBS below the established LOQ.

In the group with blood SA below 0.5 μmol/L, NTBC concentrations ranged from 3.0 to 74.3 μmol/L with a median of 28.1 μmol/L. Of these samples, only 24.8% had NTBC concentrations within the target range of 40.0–60.0 μmol/L, while 69.7% had NTBC concentrations below 40.0 μmol/L.

#### 3.2.2 Other Biomarkers

##### 3.2.2.1 Urinary δ‐ALA

**FIGURE 2:**
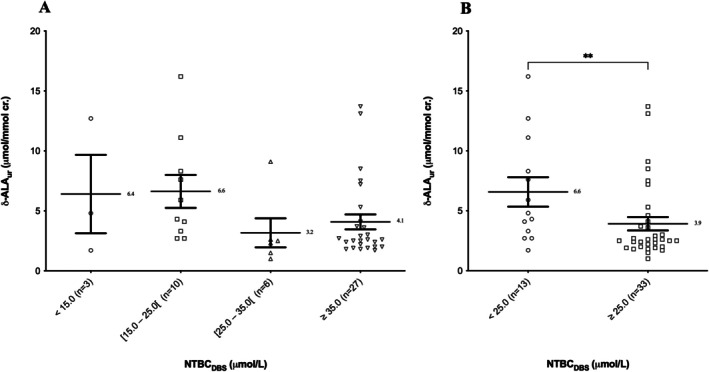
Urinary δ‐ALA concentrations according to NTBC concentrations in DBS separated in (A) four groups and (B) two groups (**p ≤ 0.01**) (mean ± SEM).

A total of 46 δ‐ALA‐NTBC/SA paired samples were collected and first classified according to their NTBC concentrations into four groups: < 15.0 (*n* = 3), [15.0–25.0 (*n* = 10)], [25.0–35.0 (*n* = 6)] and ≥ 35.0 μmol/L (*n* = 27). Despite a significant Kruskal–Wallis test (*p* = 0,0431), Dunn's multiple comparison test showed no statistically significant difference between the four groups (Figure 2A). In view of the graph obtained, the groups were re‐evaluated and separated into two distinct groups based on NTBC concentrations, namely < 25.0 μmol/L and ≥ 25.0 μmol/L. The Mann–Whitney test carried out to compare these two independent groups showed a statistically significant difference (*p* = 0.0010) (Figure 2B). Samples with an NTBC concentration below 25.0 μmol/L had a higher urinary concentration of δ‐ALA (*n* = 13; mean = 6.6 μmol/mmol creatinine (cr.)) than samples with an NTBC concentration on DBS equal or above 25.0 μmol/L (*n* = 33; mean = 3.9 μmol/mmol cr.). In fact, in the first group, 38.5% of samples were above our laboratory's threshold value (i.e., 5.5 μmmol/mol cr.) compared with 21.2% in the second group. The Spearman correlation test failed to demonstrate a correlation between NTBC concentration on DBS and urinary δ‐ALA concentration in our HT‐1 patients (*r* = −0.1990; *p* = 0.1798).

NTBC concentrations were also studied by separating samples according to our laboratory's cut‐off value for urinary δ‐ALA, that is, < 5.5 or ≥ 5.5 μmol/mmol cr., the value above which neurological repercussions may occur in patients with porphyria. The Mann Whitney test came back non‐significant (*p* = 0.2804) (Figure S1).

**FIGURE 3:**
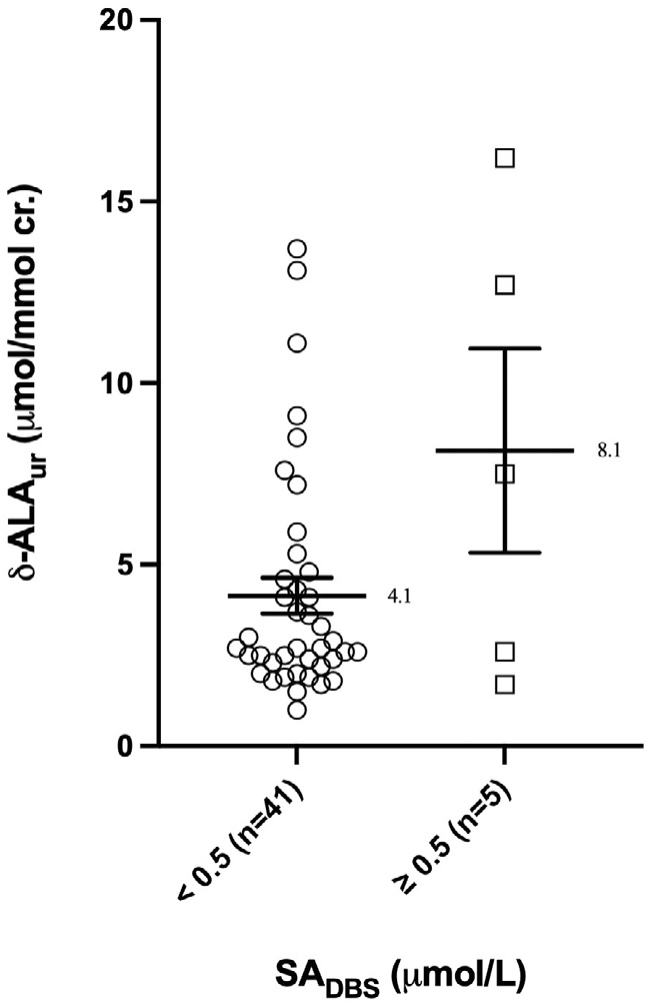
Urinary δ‐ALA concentrations according to SA concentrations in DBS separated into two groups (mean ± SEM).

The 46 paired samples were also classified according to their SA concentration in DBS. Because none of the 46 paired samples collected had a DBS SA level strictly above 1.0 μmol/L, samples were separated in two groups as follows: SA < 0.5 μmol/L (*n* = 41, mean = 4.1 μmol/mmol cr.) and ≥ 0.5 μmol/L (*n* = 5; mean = 8.1 μmol/mmol cr.) (Figure 3). The Mann–Whitney test showed no statistically significant differences between the two groups (*p* = 0.2679). A correlation test could not be performed between these two parameters as most of our paired samples had SA levels in DBS below our LOQ.

##### 3.2.2.2 AFP

AFP results measured less than 1 year after initiation of NTBC therapy were omitted. This concerned three samples from the same patient.

A total of 174 AFP‐NTBC/SA paired samples were collected and first classified according to their NTBC concentrations into four groups: < 15.0 (*n* = 40; mean = 13.9 μg/L), [15.0–25.0 (*n* = 38; mean = 8.3 μg/L)], [25.0–35.0 (*n* = 37; mean = 5.7 μg/L)] and ≥ 35.0 μmol/L (*n* = 59; mean = 6.0 μg/L) (Figure S2A). The Kruskal–Wallis (*p* = 0.1488) and Dunn's multiple comparison test showed no statistically significant differences between the four groups, even when the samples were divided into two groups (Mann Whitney test: *p* = 0.1344) (Figure S2B). NTBC concentrations were also studied by separating samples according to the threshold value classically used for AFP in HCC monitoring, that is, 10.0 μg/L. The Mann Whitney test returned nonsignificant results (*p* = 0.5059) (Figure S3). Furthermore, the Spearman correlation test failed to demonstrate the existence of a correlation between NTBC concentration on DBS and serum AFP concentration in our cohort (*r* = −0.1282; *p* = 0.1040).

The 174 paired samples were also classified according to their SA concentration in DBS in three groups: SA < 0.5 μmol/L (*n* = 144; mean = 7.4 μg/L), [0.5–1.0] μmol/L (*n* = 28; mean = 13.1 μmol/L) and > 0.5 μmol/L (*n* = 2; mean = 6.4 μmol/L) (Figure S4). The Kruskal–Wallis (*p* = 0.2822) and Dunn's multiple comparison test showed no statistically significant differences between the three groups.

### 3.3 Nutritional Monitoring

A total of 183 pairs of measurements of Phe and Tyr levels in LH plasma and DBS were collected at the same time from our 12 HT‐1 patients.

**FIGURE 4:**
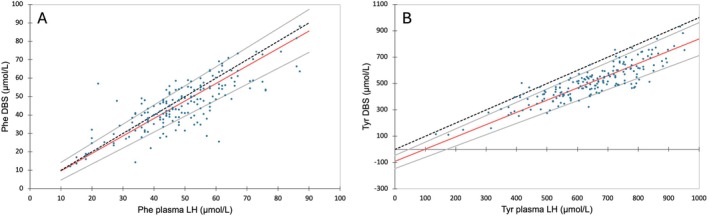
Results on Passing and Bablok fit analyses comparing (A) Phe concentrations from lithium heparin (LH) plasma and dried blood spots (DBS) and (B) Tyr concentrations from LH plasma and DBS.

The results of the Passing and Bablok regression analysis of the comparison of LH plasma versus DBS for Phe showed no difference between the two methods. On the other hand, the Passing and Bablok regression analysis for Tyr showed a lower *y*‐intercept but a normal slope, meaning there was a systematic difference between the two methods, but not a proportional one. The Passing and Bablok regression graphs confirmed these interpretations (Figure 4).

**FIGURE 5:**
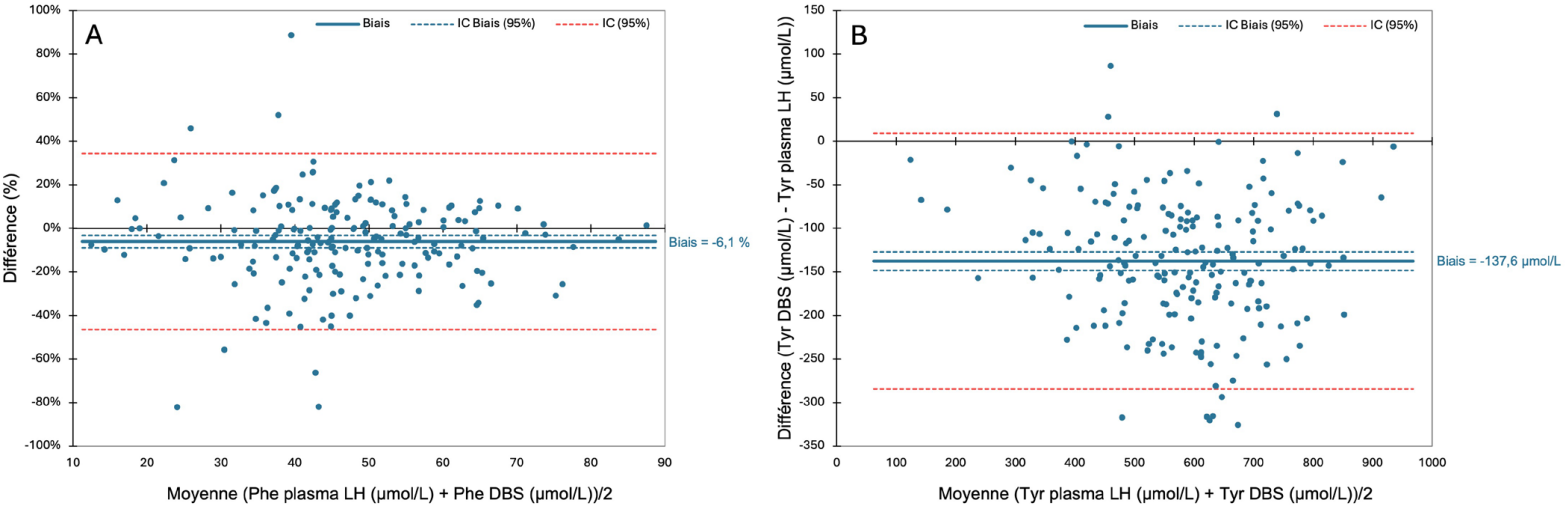
Results on Bland Altman analyses for (A) Phe concentrations measured in lithium heparin (LH) plasma compared to dried blood spots (DBS) and (B) Tyr concentrations measured in LH plasma compared to DBS.

Bland–Altman analyses were also performed. Following the results obtained by the Passing and Bablok regression analysis, the percentage differences in Phe concentrations between the two methods were compared with their mean concentrations (Figure 5A), while the absolute differences in Tyr concentrations between the two methods were compared with their mean concentrations due to the constant difference between the two methods (Figure 5B).

Phe concentrations obtained on DBS were on average 6.1% (±20.6%) lower than those obtained on LH plasma. For the Bland–Altman of Tyr, while there is a good distribution of points around the mean bias in the 400–800 μmol/L range, the points outside this range appear to be distributed on one side of the bias. This could suggest a proportional bias in these regions (< 400 μmol/L and > 800 μmol/L). Unfortunately, we did not have enough data in these concentration ranges to perform statistical analysis. Tyr concentrations obtained on DBS were on average 137.6 μmol/L (±75.0 μmol/L) lower than those obtained on LH plasma. However, caution should be exercised with values outside the 400–800 μmol/L range.

## 4 Discussion

The prognosis of patients with HT‐1 has improved considerably since the introduction of NTBC in 1992 due to its ability to successfully reduce the production of toxic metabolites and thereby resolve liver failure and renal tubulopathy and prevent pseudo‐porphyric acute crisis. Until the 2010s, therapeutic monitoring of NTBC treatment in HT‐1 patients was generally performed by measuring urinary SA excretion and measuring Tyr and Phe concentrations in serum or plasma. However, since then, numerous methods have been published for determining NTBC concentrations in serum, but also in DBS, with good correlation between the two matrices [15, 16, 18, 19, 22, 23, 24, 25, 26, 27]. This has led to the introduction of target values for NTBC in the latest published recommendations [4, 8]. In addition, the marked improvement in the sensitivity of the latest analytical methods has made it possible to measure SA in blood, which is also included in these guidelines. Unfortunately, there is currently no available evidence to support one monitoring approach over another, and only a very limited number of publications reporting experience with either monitoring method [15, 18, 19]. Furthermore, although the guidelines emphasise monitoring AFP levels, no clear information exists regarding urinary δ‐ALA levels [4, 8, 20, 28]. Therefore, the optimal global monitoring strategy remains debated.

In this study, we aimed to present our single‐centre experience and thus attempt to evaluate the clinical utility of monitoring NTBC concentration and to determine whether it provides added value compared to other biological markers classically used in the monitoring of HT‐1 patients treated with NTBC. We also aimed to evaluate the usefulness of DBS samples in this context. To this end, we retrospectively analysed the biological data of 12 HT‐1 patients followed at the Nutrition and Metabolism Unit of HUDERF in Brussels, Belgium, over a 6‐year period.

Firstly, our results showed that NTBC concentrations were significantly higher in samples with a blood SA concentration that could not be quantified by our method (i.e., < 0.5 μmol/L) than in samples with a quantifiable blood SA concentration (i.e., ≥ 0.5 μmol/L). In addition, a wide range of NTBC concentrations in our cohort corresponded to normal SA values in the blood (< 0.5 μmol/L) and were undetectable in the urine. Our data indicate that a broad range of NTBC concentrations is sufficient for SA suppression, thereby challenging the relevance of current NTBC target values. Consequently, SA levels appear to be a more reliable indicator of adequate metabolic control than NTBC concentrations.

One of the well‐known complications of HT‐1 is the occurrence of acute neurological pseudo‐porphyric seizures following inhibition of δ‐ALA dehydratase by SA and hence the accumulation of δ‐ALA [29]. Our study was the first to investigate correlations between blood levels of NTBC and SA with urinary δ‐ALA levels. At first, the separation of samples according to NTBC concentrations in four groups gave a significant Kruskal–Wallis test but Dunn's post‐test came back inconclusive. This could be explained by the large difference in size between groups. When separated in two groups, the results in our cohort showed significantly higher levels of δ‐ALA in samples with NTBC concentrations below 25.0 μmol/L (mean = 6.6 μmol/mol cr.) compared with samples with NTBC concentrations greater than 25.0 μmol/L (mean = 3.9 μmol/mol cr.). Unfortunately, no clear correlation could be demonstrated between urinary δ‐ALA and NTBC concentrations in our cohort. However, a higher level of urinary δ‐ALA could indicate residual production of toxic metabolites and therefore incomplete inhibition of the metabolic pathway by NTBC. Our results seem to suggest that adapting individual NTBC concentrations to reach values of 25.0 μmol/L or higher would ensure better inhibition of the metabolic pathway, a value below the currently recommended concentrations. This seems to be in line with our conclusions based on blood SA levels. Moreover, due to their mechanistic link, SA measurement should in theory be a good reflection of δ‐ALA accumulation. However, no statistically significant difference was found between the two groups (SA < 0.5 μmol/L and SA ≥ 0.5 μmol/L). In addition, eight samples from four patients presented urinary δ‐ALA levels above our laboratory cut‐off value (i.e., 5.5 μmol/mmol cr.) despite having SA levels in DBS below 0.5 μmol/L. Our findings could suggest that SA measurements are not enough to monitor the risk of acute pseudo‐porphyric crisis. But this could also be explained by the large difference in size between the two groups and further research is needed.

HCC is another significant complication of HT‐1 [30]. Currently, AFP is the sole biochemical marker used to diagnose this cancer. Recent publications have reported the development of HCC despite NTBC therapy and normalisation of AFP in three patients [31]. Furthermore, SA measurements alone cannot conclusively verify complete pathway blockade or exclude intracellular production of FAA—the most hepatotoxic metabolite. Identifying an earlier parameter to prevent HCC in these patients would therefore be valuable. However, statistical analysis within our cohort did not reveal any correlation between blood NTBC levels and AFP, which aligns with findings reported by Fuenzalida et al. [15]. Consequently, while AFP remains clinically relevant, it appears unrelated to NTBC blood concentration, and imaging continues to be the gold standard for monitoring the occurrence of HCC in HT‐1 patients.

All our patient samples had urinary SA levels below the quantification limit, even in those with δ‐ALA and AFP above cut‐off values. This suggests blood SA measurement may be superior to urine SA excretion for long‐term HT‐1 monitoring, as it avoids issues related to urinary concentration and creatinine adjustment and may allow quicker detection of SA side effects or facilitate NTBC dosage adjustments. Further study on a larger group of NTBC‐treated HT‐1 patients is needed.

Overall, our findings suggest that NTBC measurement may have a more restricted role in the long‐term monitoring of HT‐1 patients than previously assumed. Although NTBC remains essential as a therapeutic agent, its blood concentration does not consistently predict or reflect metabolic control, nor does it correlate reliably with the traditional biomarkers used to assess disease activity or complications. In contrast, SA presents a more direct and reliable marker of pathway inhibition, as SA suppression was observed across a broad range of NTBC concentrations and was more closely aligned with metabolic stability than NTBC itself. Nevertheless, neither NTBC nor SA alone fully captured the risk of neurological complications, as urinary δ‐ALA elevations were detected even when SA was unquantifiable, highlighting the need to continue δ‐ALA assessment in selected clinical contexts. Similarly, while AFP remains crucial for HCC surveillance, it showed no relationship to NTBC concentration, reaffirming the necessity of AFP assessment and imaging in hepatic monitoring. Taken together, these results support a monitoring strategy centred primarily on SA, complemented by δ‐ALA and AFP for the evaluation of acute neurological risk and HCC development, while suggesting that NTBC concentration should be interpreted cautiously and may offer limited added value beyond ensuring treatment adherence, interpreting unexplained SA elevation, and avoiding excessively low or high exposure.

However, our conclusions should be treated with caution, since our study is subject to some limitations, including a small number of patients and samples, non‐uniformity of sampling time with respect to NTBC intake, and the absence of an indirect compliance study. In fact, we observed considerable variability in NTBC concentrations despite identical dosages between certain patients. Unfortunately, it is impossible to know whether these differences are linked to poor patient compliance, inter‐individual metabolism of NTBC, or problems with the standardisation of sampling.

In the context of nutritional monitoring, we studied the correlations between Tyr and Phe concentrations in DBS and plasma. For Phe, we observed lower concentrations in DBS than in plasma with an average constant bias of 6.1%, consistent with most previous studies, regardless of the anticoagulant used [21, 32, 33, 34]. In the case of Tyr, our results showed a much greater difference between the two methods. Tyr showed larger and more variable differences between DBS and plasma. Previous reports on Tyr are limited and inconsistent. While Allard et al. found no difference, Groselj et al. reported 15.5% lower DBS concentrations [32, 33]. Moreover, our results tend to demonstrate the potential difficulty of establishing a correction factor between DBS and plasma for Tyr, whereas that is what recently published guidelines propose [35]. These discrepancies can be partly explained by AA transport mechanisms: Phe, transported via the L‐type system, does not accumulate in erythrocytes, leading to lower DBS concentrations, while Tyr uses both L‐type and A‐type systems, resulting in more variable erythrocytes/plasma ratios [36]. Methodological differences (calibration, extraction, detection) and individual factors such as haematocrit, blood volume applied, and sample transport can further contribute to variability [21, 34]. Despite the logistical advantages of DBS, plasma remains the preferred method for Tyr and Phe monitoring. If DBS is nevertheless used in this context, plasma confirmation is recommended when DBS concentrations approach critical values.

In conclusion, this single‐centre study highlights the strengths and limitations of currently available biomarkers in the long‐term monitoring of HT‐1 patients treated with NTBC. While NTBC therapy remains indispensable, its blood concentration seems not to reliably indicate metabolic control, neurological risk, or hepatic complications. In contrast, SA suppression proved to be a more reliable marker of therapeutic efficacy, as its suppression was consistently achieved across a broad spectrum of NTBC concentrations. Nevertheless, SA alone seems to be insufficient to fully assess neurological risk, making urinary δ‐ALA still relevant in certain cases. Similarly, AFP and imaging remain essential for early detection of HCC and cannot be replaced by NTBC monitoring. Together, these data support a monitoring strategy that prioritises SA measurement, complemented by δ‐ALA and AFP according to clinical context, while reserving NTBC quantification for assessing compliance, unexplained SA elevation, or extreme exposure. Finally, AA monitoring via DBS remains challenging, especially for Tyr, reinforcing plasma as the preferred matrix. Larger, multi‐centre studies are needed to confirm these findings and to refine evidence‐based recommendations for the optimal biochemical follow‐up of patients with HT‐1.

## Author Contributions

**Anne‐Sophie Adam:** study design, data collection, data analysis, manuscript writing, manuscript editing. **Lionel Marcélis:** study design, data collection, data analysis, manuscript editing. **David Fage:** data analysis, manuscript writing, manuscript editing. **Elise Mathieu:** manuscript writing, manuscript editing. **Aurélie Empain:** manuscript writing, manuscript editing. **Céline Dufour:** manuscript writing, manuscript editing. **Frédéric Cotton:** manuscript writing, manuscript editing. **Corinne de Laet:** study design, data collection, data analysis, manuscript writing, manuscript editing. A.‐S. Adam serves as guarantor for the article, accepts full responsibility for the work and/or the conduct of the study, has access to the data, and controlled the decision to publish. All authors have accepted responsibility for the entire content of this manuscript and approved its submission.

## Funding

The authors have nothing to report.

## Ethics Statement

All human research conducted was in accordance with the required ethical standards and was approved by the HUDERF Ethics committee (granted on 30/05/2024; CEH 39/24).

## Conflicts of Interest

The authors declare no conflicts of interest.

## Supporting information


**Data S1:** Supporting Information.

## Data Availability

The data that support the findings of this study are available from the corresponding author upon reasonable request.
