# Supplementary material for: Therapeutic Monitoring of Patients With Hereditary Tyrosinemia Type 1—A Belgian Monocentric Experience
Source: JIMD Rep. 2026 Jan 7;67(1):e70062. doi: 10.1002/jmd2.70062 (PMC12779275; doi:10.1002/jmd2.70062)
Supplement: Supplementary file 1 — Data S1: Supporting Information. [file JMD2-67-e70062-s001.docx]

**Supplementary Materials**

Table S1: Laboratory follow-up parameters of our 12 HT-1 patients (median, mean, minimum-maximum).

| **Patients** | **Analysis** | **1** | **2** | **3** | **4** | **5** | **6** | **7** | **8** | **9** | **10** | **11** | **12** |
| --- | --- | --- | --- | --- | --- | --- | --- | --- | --- | --- | --- | --- | --- |
| **DBS** |  |  |  |  |  |  |  |  |  |  |  |  |  |
| **NTBC** (µmol/L) | Median Mean ± SD Min-Max (N) | 15.0 18.0 ± 7.5 7.0 - 37.2 (17) | 33.5 36.4 ± 16.2 13.4 - 65.3 (16) | 17.7 23.1 ± 13.8 3.1 - 53.2 (19) | 36.6 38.3 ± 11.8 23.1 - 59.8 (14) | 45.5 45.3 ± 19.6 15.1 - 74.3 (12) | 25.0 28.4 ± 18.6 4.9 - 62.4 (19) | 23.4 27.8 ± 17.4 9.6 - 63.9 (16) | 27.1 26.5 ± 11.2 10.8 - 49.1 (19) | 34.3 34.0 ± 12 13.6 - 49.7 (15) | 34.6 39.9 ± 19.4 14.3 - 82.8 (17) | 21.2 21.5 ± 11.4 4.3 - 43.7 (15) | 16.7 20.9 ± 15.6 1.1 - 48.0 (18) |
| **SA** (µmol/L) | Median Mean ± SD Min-Max (N) | X X <0.5 - 1.6 (17) | X X All <0.5 (16) | X X < 0.5 - 1.1 (18) | X X All < 0.5 (14) | X X < 0.5 - 1.0 (12) | X X < 0.5 - 0.7 (19) | X X <0.5 - 0.6 (16) | X X <0.5 - 0.8 (19) | X X < 0.5 - 0.9 (15) | X X <0.5 - 0.9 (17) | X X <0.5 - 0.6 (15) | X X < 0.5 - 1.2 (18) |
| **Phe** (µmol/L) | Median Mean ± SD Min-Max (N) | 30 34 ± 15 6 - 59 (17) | 42 43 ± 10 23 - 58 (16) | 45 45 ± 9 28 - 61 (19) | 41 38 ± 12 16 - 56 (14) | 48 49 ± 9 38 - 71 (12) | 42 42 ± 11 22 - 72 (19) | 56 61 ± 11 46 - 89 (16) | 43 46 ± 11 31 - 74 (19) | 38 36 ± 16 12 - 60 (15) | 40 38 ± 10 14 - 55 (17) | 59 60 ± 14 38 - 88 (15) | 57 59 ± 15 46 - 114 (18) |
| **Tyr (µmol/L)** | Median Mean ± SD Min-Max (N) | 362 342 ± 120 91 - 590 (17) | 482 489 ± 107 296 - 755 (16) | 570 547 ± 163 257 - 932 (19) | 489 527 ± 105 387 - 695 (14) | 544 528 ± 77 336 - 616 (12) | 511 538 ± 160 273 - 825 (19) | 611 610 ± 73 488 - 738 (16) | 507 535 ± 110 402 - 838 (19) | 417 417 ± 101 251 - 599 (15) | 434 469 ± 86 344 - 685 (17) | 660 640 ± 114 465 - 882 (15) | 547 570 ± 140 418 - 946 (18) |
| **Plasma/Serum** |  |  |  |  |  |  |  |  |  |  |  |  |  |
| **AFP (µg/L)** | Median Mean ± SD Min-Max (N) | 5.2 7.9 ± 8.7 3.0 - 32.0 (10) | 5.1 5.1 ± 0.7 4.1 - 6.3 (8) | 3.4 15.8 ± 33.8 3.0 - 121.0 (15) | 3.2 5.3 ± 7.4 3.0 - 31.0 (14) | 7.6 7.6 ± 1.2 5.2 - 10.8 (12) | 3.1 14.6 ± 32.6 2.3 - 135.0 (18) | 4.6 4.5 ± 0.2 4.0 - 4.8 (16) | 3.3 3.2 ± 0.5 2.3 - 4.3 (19) | 5.7 5.6 ± 0.6 4.3 - 6.7 (15) | 9.3 9.3 ± 0.7 8.6 - 10.9 (14) | 8.9 9.1 ± 0.6 8.2 - 10.2 (15) | 9.2 9.4 ± 0.8 8.0 - 10.9 (18) |
| **Phe (µmol/L)** | Median Mean ± SD Min-Max (N) | 44 46 ± 18 19 - 76 (13) | 46 47 ± 16 22 - 76 (16) | 46 45 ± 6 32 - 59 (18) | 44 41 ± 13 18 - 63 (13) | 50 53 ± 10 43 - 71 (10) | 43 45 ± 12 31 - 81 (18) | 61 64 ± 16 45 - 113 (16) | 48 48 ± 9 33 - 73 (19) | 33 34 ± 17 13 - 65 (14) | 40 42 ± 13 15 - 72 (17) | 62 64 ± 15 42 - 87 (15) | 52 60 ± 11 45 - 87 (13) |
| **Tyr (µmol/L)** | Median Mean ± SD Min-Max (N) | 469 479 ± 144 225 - 756 (13) | 631 641 ± 87 510 - 795 (16) | 682 679 ± 151 394 - 938 (18) | 718 689 ± 100 531 - 852 (13) | 714 697 ± 106 441 - 800 (10) | 716 700 ± 164 382 - 954 (18) | 736 750 ± 72 633 - 881 (16) | 671 679 ± 99 529 - 862 (19) | 490 511 ± 107 308 - 669 (14) | 553 601 ± 165 417 - 1105 (17) | 794 792 ± 98 605 - 947 (15) | 703 704 ± 102 543 - 918 (13) |
| **Urine** |  |  |  |  |  |  |  |  |  |  |  |  |  |
| **d-ALA (µmol/mol cr.)** | Median Mean ± SD Min-Max (N) | 8.3 8.3 8.3 (1) | 3.3 3.3 ± 0.6 2.7 - 4.1 (6) | 12.7 12.5 ± 3.9 8.5 - 16.2 (3) | 1.9 37.8 ± 62.3 1.8 - 109.8 (3) | 2.4 2.3 ± 0,4 1.9 - 2.6 (3) | 2.2 2.2 ± 0.6 1.5 - 2.9 (4) | 1.9 2.0 ± 0.4 1.7 - 2.7 (6) | 4.3 3.8 ± 1.1 2.6 - 4.6 (3) | 2.4 2.0 ± 0.7 1.0 - 2.5 (4) | 7.6 9.1 ± 3.2 5.3 - 13.7 (7) | 3.3 3.5 ± 1.2 2.5 - 4.8 (3) | 4.1 4.2 ± 1.6 2.6 - 5.9 (3) |

Figure S1: NTBC concentrations according to our laboratory's urinary δ-ALA cut-off concentration for possible neurological repercussions (mean±SEM).


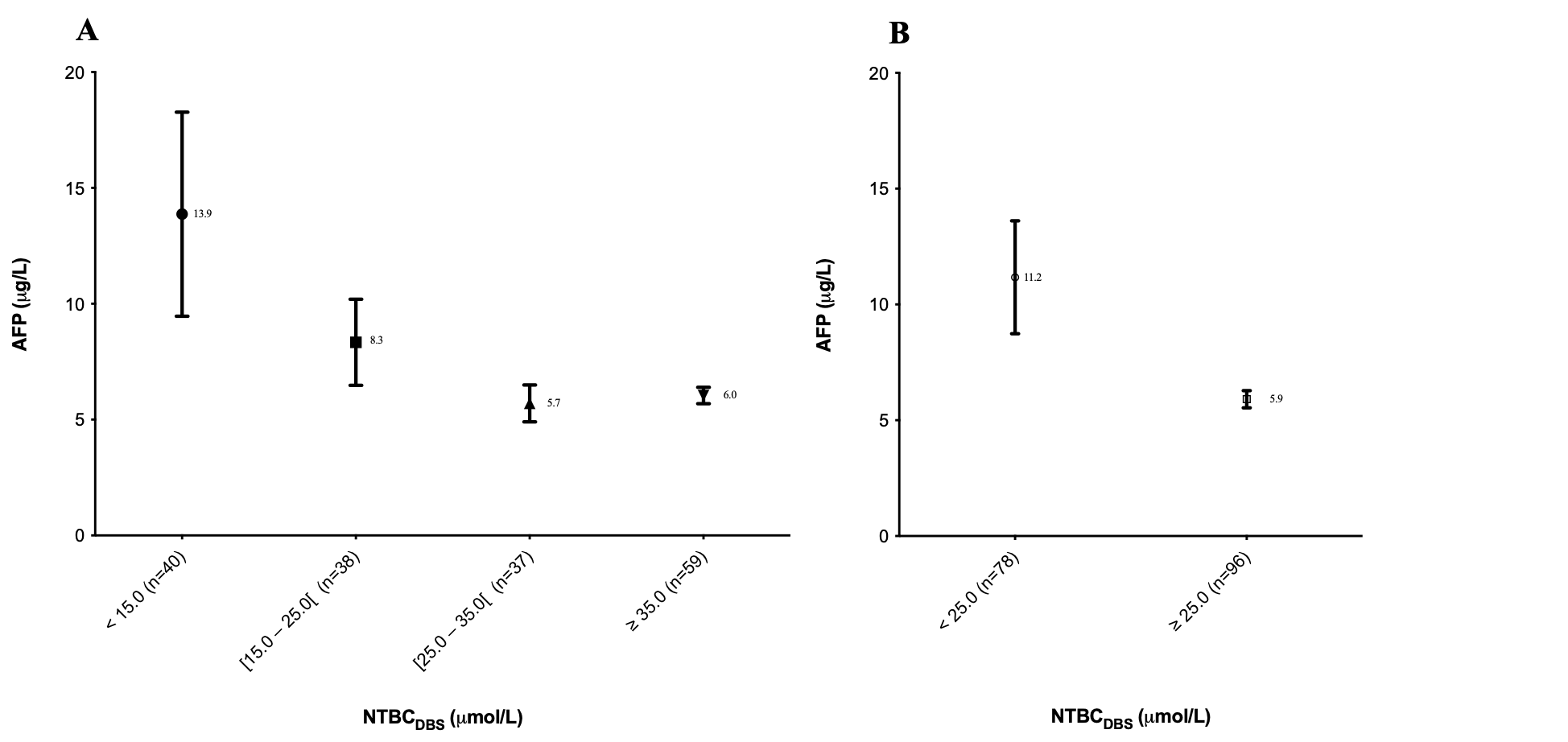


Figure S2: Serum AFP concentrations according to NTBC concentrations in DBS separated in

(A) four groups and (B) two groups (mean±SEM).

Figure S3: NTBC concentrations according to serum AFP cut-off concentration for hepatocellular carcinoma (mean±SEM).

Figure S4: Serum AFP concentrations according to SA concentrations in DBS separated in

three groups (mean±SEM).

### **NTBC, Phe, Tyr and SA assays on DBS**

#### Material and reagents

SA, hydrazine monohydrate, butanol, hydrochloric acid (HCl), trifluoroacetic acid (TFA) and formic acid (FA) were purchased from Sigma Aldrich (Merck - Darmstadt, Germany). NTBC was purchased as the pharmaceutical speciality Orfadin® (Sobi, Sweden). Internal standards were purchased from Cambridge Isotope Laboratories (amino acid mix A NSK-A, labelled SA NSK-T-1) and Sigma-Aldrich (mesotrione). HPLC-grade solvents (methanol, acetonitrile and water) were purchased from Biosolve. Other consumables, including the 96 well microplates, were purchased from VWR. The Perkin Elmer 226 filter paper for newborn screening was purchased from Revvity.

#### Sample preparation

For NTBC analysis in DBS, a 3.2 mm disc was punched to a flat-bottom 96-well plate. Methanol (200 μL) containing mesotrione (1 µmol/L; internal standard) was added to each well. The mixture was shaken for 30 min at room temperature (RT). The supernatants were transferred to another 96 well microplate vials and injected into the LC-MS/MS system. A conversion factor between DBS and plasma of 2.34 was directly applied to each NTBC result, as described in a previous publication of our laboratory and in order to be able to compare our results with the blood concentrations included in the recommendations [14].

For amino acids (AA) analysis in DBS, a 3.2 mm disc was punched and transferred to a flat-bottom 96-well plate. DBS were extracted with 200 μL of methanol. The plate was sealed and eluted without agitation at RT for 20 min. Then, 40 μL of each supernatant was transferred to a 96-well plate and 160 μL of methanol containing isotopically labelled AA internal standard (NSK-A) was added to each well. The samples were evaporated to dryness under a stream of nitrogen. Butylation was carried out by resuspending the dry extract with 25 µL of butanol-HCl 3N and incubating at 65 °C for 20 min. The samples were evaporated to dryness and resuspended in 200 µL of ACN 20% and 0.05% FA and then injected into the LC–MS/MS system.

For SA analysis in DBS, the 3.2 mm disc eluted by methanol for AA analysis was dried and further extracted with 200 µL of 80% acetonitrile, 20% water containing 0.1% formic acid (v/v), hydrazine monohydrate (20 mmol/L) and isotopically labelled SA internal standard (NSK-T; 0.5 mmol/L). The plate was sealed and eluted by gently mixing at 450 rpm for 40 min at 50°C. After incubation, the supernatants were transferred to a 96-well plate and injected into the LC-MS/MS system.

Each run of either three methods used an internal control and calibration curve.

#### Instrumentation and analysis

NTBC and SA determinations were performed using an API 4000 LC-MS/MS system (AB SCIEX, Framingham, MA USA).

For NTBC quantification, the chromatographic system was equipped with a Phenomenex Gemini® 3 µm NX-C18 column 110 Å. Chromatographic separation of NTBC was performed using an isocratic flow of 0.2 mL/min with a mobile phase comprising 60% ACN, 0.1% FA and 0,001% TFA and thermostated at 40 °C. The sample volume injection was 5µL. MS/MS detection of NTBC was through multiple reaction monitoring (MRM) in positive electrospray ionization mode. The quantifier and qualifier transitions for NTBC were 330/218 and 330/126 m/z, respectively. For mesotrinone, it was 340/228 and 340/104 m/z.

AA determinations were performed using an API 3200 LC-MS/MS system (AB SCIEX, Framingham, MA USA) and by injecting 20 µL of sample directly into the MS/MS detector using an isocratic flow of 0.08 mL/min with a mobile phase comprising 20% ACN and 0.1% FA. The ESI–MS/MS parameter was optimized for each butylated AA and its corresponding labeled internal standard. The AA concentrations were determined with MRM mode.

For SA determination, 5 µL of sample was injected directly into the MS/MS detector using an isocratic flow of 0.2 mL/min with a mobile phase comprising 80% ACN and 0.05% FA. The sample volume injection was 5 µL. MS/MS detection of SA was through MRM in positive electrospray ionization mode. The quantifier transitions for SA were 155/137 m/z and for SA-C5, 160/142 m/z.

Accuracy for each analyte assayed was determined by analyzing external samples from ERNDIM (European Research Network to Evaluate and Improve Screening, Diagnosis, and Treatment of Inherited Disorders of Metabolism). The limits of quantification were as follows: NTBC 0.5 µmol/L; SA 0.5 µmol/L; Phe 1.02 µmol/L and Tyr 1.36 µmol/L.
